# Symbolic Metal Bit and Saddlebag Fastenings in a Middle Bronze Age Donkey Burial

**DOI:** 10.1371/journal.pone.0058648

**Published:** 2013-03-06

**Authors:** Guy Bar-Oz, Pirhiya Nahshoni, Hadas Motro, Eliezer D. Oren

**Affiliations:** 1 Zinman Institute of Archaeology, University of Haifa, Haifa, Israel; 2 Department of Bible, Archaeology and Ancient Near Eastern Studies, Ben-Gurion University, Beer-Sheva, Israel; 3 Koret School of Veterinary Medicine, Hebrew University of Jerusalem, Rehovot, Israel; Universidad Autonoma de Barcelona and University of York, Spain

## Abstract

Here we report the unprecedented discovery of the skeleton of a ritually interred donkey with a metal horse bit in association with its teeth and saddlebag fastenings on its back. This discovery in the Middle Bronze Age III sacred precinct (1700/1650-1550 BCE) at Tel Haror, Israel, presents a unique combination of evidence for the early employment of equid harnessing equipment, both for chariot bridling (horse bit) and pack animals (saddlebags). The ritually deposited donkey with its unique accoutrements advances our understanding of the broad social and religious significance of equids in the Levantine Bronze Age, previously known mainly from textual and iconographical sources.

## Introduction

Ritual burial of equids is an intriguing aspect of the ritual life of third and second millennium BCE societies in Eurasia and North Africa. Well-known examples in the Levant and Egypt include donkey burials from Early Dynastic Egypt (e.g., Abydos, Tarkhan, Abusir, and Helwan; cf. [1]) and in Early Bronze Age Syria and Iraq (e.g., Tell Halawa, Tell Brak, Umm el-Mara, Abu Salabikh, Kish and Ur; e.g., [2]-[4]). These interments have been variously interpreted, as reflections of the funerary ideology of the elite, ancestor cults, and temple sacrifice. This phenomenon reached its peak in the Levant and Egypt in the beginning of the second millennium BCE in conjunction with the emergence of urban societies, large-scale trade and highly organized warfare [5].

Archaeological contexts in which equid remains are known during the Levantine Middle Bronze Age (MBA) include equid burials in ritual and mortuary settings, which point strongly at their symbolic and religious significance among MBA societies. Numerous examples of donkey burials are associated with warriors or, presumably, high-ranking individuals in MBA tombs from the southern Levant (Tell el-Ajjul, Lachish, Akko, Jericho [6], [7]) and the eastern Nile Delta (Tell el-Dab'a, Tell el-Maskhuta, Inshas [8], [9]). A number of Levantine tombs contained equids, interred singularly, in pairs, or in larger groups. In some cases, disarticulated bones and taphonomic evidence for ritual slaughter and consumption, presumably testify to their use in funerary feasts and ceremonial deposition [6], [10]–[11]. Equid interments in Egypt often involve the deposition of pairs of donkeys inside and/or in front of the entrances to tombs [8], [12]–[15]. At the MBA Syrian site of Umm el-Marra complete equids were ritually deposited in a circular shaft on the acropolis [4], [15]. In other examples equids were found buried under walls as foundation deposits or in simple pits [6]. Those equid remains from burial contexts that were subject to systematic archaeozoological study were shown to belong to donkeys and, less frequently, to other equids, including, possibly, donkey-onager hybrids [4], [6].

Here, we report the discovery of an articulated skeleton of a young donkey (*Equus asinus*) from MBA Tel Haror in the western Negev, Israel. This donkey was ritually deposited inside a specially constructed installation in the city's sacred precinct, near a diagnostic Syrian-type temple [16]. Owing to the relatively arid conditions of the site, the well-preserved burial included the unique occurrence of a donkey being bridled with the mouthpiece of a metal horse bit in its mouth and the metal remains of saddlebags on its back. These finds represent a unique combination of evidence for the early use of equid harnessing equipment both for chariot bridling (horse bit) and pack animals (saddlebags) in the ancient Near East. The Tel Haror interment represents the only known example of a donkey within a ritual context that was symbolically harnessed with a horse bit and bearing saddlebags, and, thus, sheds important light on both the functional and symbolic role of equids in the Ancient Near East.

## Results

### Archaeological Context of the Donkey Interment

**Figure 1:**
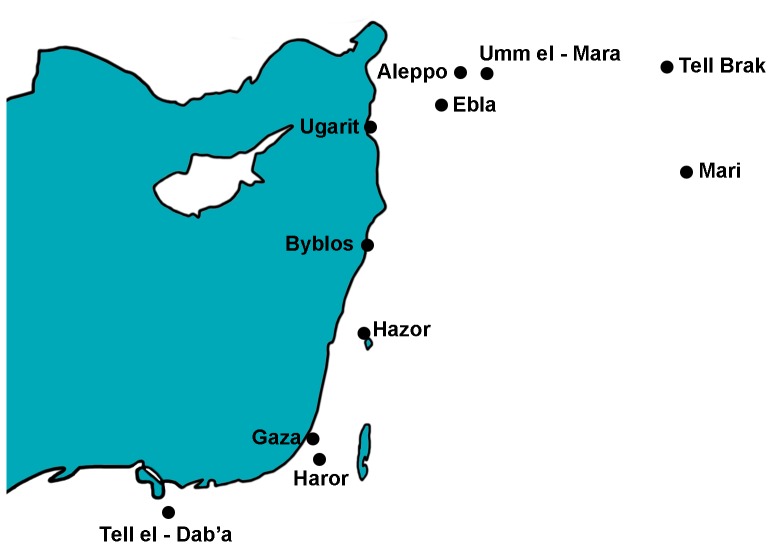
Location map showing Tel Haror and other sites mentioned in the text.

Tel Haror is one of the largest (ca. 40 acres) Bronze Age sites in southern Israel and is situated ca. 20 km east of the Mediterranean Sea (Figure 1). Excavations at the site uncovered substantial remains of an MBA III (1700/1650-1550 BCE) city that was fortified by massive ramparts and a deep moat. A sacred precinct in the southwestern corner of the city (Area K; Figures S1 and S2), which spanned three strata (VI-IV), includes the remains of a Syrian-type temple, a storehouse or temple magazine, a spacious courtyard with offering altars and numerous cultic repositories, and auxiliary service structures [17]. Among the cultic characteristics of the precinct throughout its existence, are the remains of numerous sacrifices, such as complete skeletons of young dogs and birds [18], some of which are associated with clay figurines and miniature vessels, all indicating that the area functioned continuously as a religious precinct [16].

**Figure 2:**
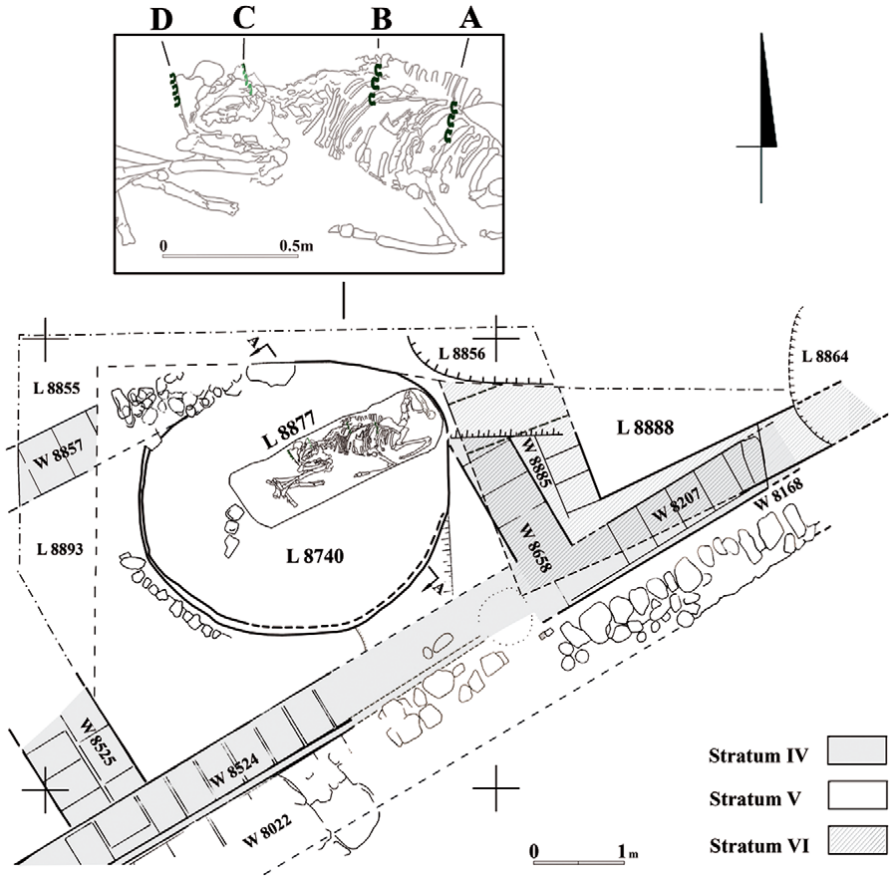
Plan of the offering installation and donkey interment with close-up of groups of saddlebag fasteners.

**Figure 3:**
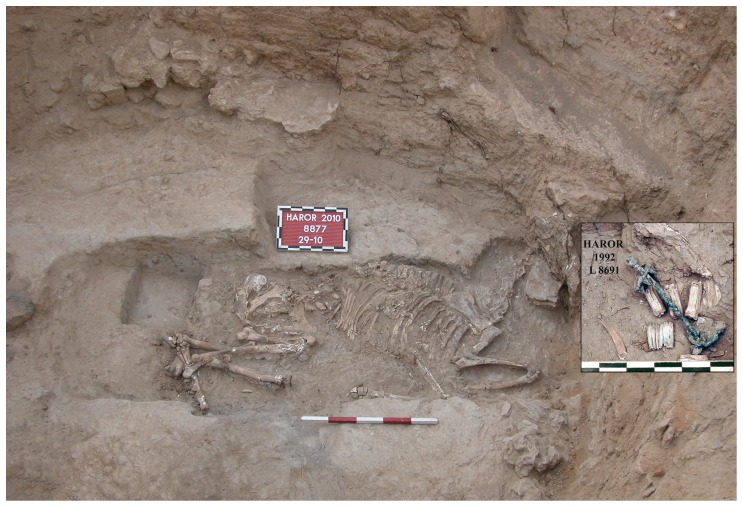
The donkey interment inside the circular installation. Notice the 1992 find of the donkey's skull and bit *in situ* (on the right).

The donkey interment was discovered within a Stratum V installation adjacent to a 3.0×6.0 m rectangular offering chamber with two narrow benches for offerings and recessed niches in its walls (Figure 2, Figure S2). The installation is roughly circular (3.5 m long, 3 m wide and at least 1.8 meters high) and both cuts through an earlier stratum and the hewn *kurkar* bedrock (Figure S3); its sides were coated with a thick layer of mud plaster. The installation was filled with a thick stratified accumulation of ash, hearths and animal bones, all of which testifying to continuous ritual activity involving the burning and deposition of sacrificial animal remains. In a later phase of Stratum V, this accumulation was cut by an elongated burial pit (0.8×2.4 m and 0.9 m deep) in which the donkey was found fully articulated (Figure 3).

The donkey had been carefully laid on its left side with its head, somewhat elevated, leaning against the wall of the pit and with three of its limbs neatly bent (Table S1). The left hind leg seems to have been intentionally broken, perhaps after rigor mortis, so as to fit it into the pit. Dental wear and eruption and bone fusion of the donkey indicate that it was a young individual, approximately 4 years old. This specimen also lacked any exceptional pathological or taphonomic modification such as butchery marks or burning bones. However, the most outstanding feature of this burial was the discovery that the donkey was bridled with a metal bit in its mouth and with the metal fasteners of saddlebags on its back.

Another skeleton, partially disarticulated, with a missing skull, belonging to an older donkey was found directly on top of the young donkey's hind section (Table S1). A layer of distinctive reddish-brown clay sediment covered both donkeys and was topped by an ash layer containing additional bones of butchered sheep and goat (L8871). The fill deposit of the burial pit above (L8738) consisted of light gray sediment with an ash lens mixed with sheep and goat bones (L8741). An ash deposit sealed the burial pit and covered the entire offering installation (L8745). An additional mandible of a donkey was found above L8745 along with the upper part of a storage jar and a large section of a bowl (L8746), all of which were covered by a thick layer of reddish-brown sediment (L8730; Figure S4). Later building operations above the offering installation have damaged its topmost part. However, the preserved segments of the thick mud plaster along the inner brim of the circular installation indicate that it originally had a distinctively dome-shaped cover.

The dating of the donkey burial at Haror to the MBA is based on the site stratigraphy, analysis of pottery assemblages found in association with the interment chamber, and correlations with well-established regional ceramic sequences grounded in widely accepted relative and absolute chronologies. Ceramic chronological markers from the burial installation include restorable cult vessels and small favissae diagnostic of the MBA III period in the southern Levant (1700/1650-1550 BCE; described in [16]: 252–283). The presence of local and imported wares within the deposit establishes links with the late 2^nd^ intermediate period in Egypt and Middle Bronze Age in Syria that provide a restricted chronological range. Direct radiometric dating of the donkey skeleton and associated finds was not possible due to the poor preservation of bone collagen and the dearth of datable organic material; a situation broadly characteristic of MBA sites in this region (e.g., [19]).

### Symbolic Bit in the Donkey's Mouth

**Figure 4:**
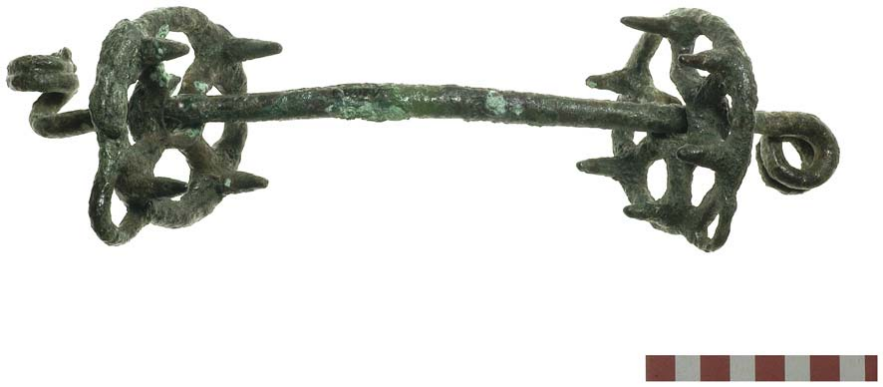
The bridle bit (Specimen IAA # 2009-951, The Israel Museum, Jerusalem).

The copper bridle bit was made of a solid-forged, round-sectioned (1.1 cm) bar mouthpiece and a pair of cast discoid cheek pieces that are studded on their inner face (Figure 4 and Figure S5; [16], [20]; Specimen IAA # 2009-951, The Israel Museum, Jerusalem). The 6.3 cm diameter cheek-pieces are ‘spoke-wheeled’ and include loops for the attachment of the cheek straps of the headstall and to the central hole for the mouthpiece bar. It should be noted that the two cheek pieces are not a matching pair. The number of studs correspond to the number of ‘spokes’, four and six, respectively, implying that the bit was assembled from three individual parts that did not originally belong together. The long mouthpiece (24.0 cm) is finished at both ends by thinning out the bar to ca. 0.7 cm and then twisting them to form spiraled loops for attaching the reins either directly or by means of rings (e.g., [21]: Figure 46a). Judging from the size of the spirals, both cheek-pieces seem to have been mounted after the bending of the first spiral and then the opposite spiral was shaped. Most known examples of this bit type terminate in loops that roll back on themselves and in the same plane ([22]: Figure 1, Pl. XII). Compared with bits from later periods and modern examples ([23]:168–9), the mouthpiece from Tel Haror is exceptionally long. The combination of the long mouthpiece with the studded cheek-pieces would have provided effective leverage that could improve directional control and maneuverability of the animal. These characteristics are important in horse bridling, especially when used in a draught team pulling a chariot ([23]:168–9, [24]).

It is likely that the reassembled Haror bit was already quite defective when placed in the mouth of the interred donkey and was no longer an effective steering device. This supposition is supported by the presence of a distinctive gap in both collars of the cheekpieces resulting from extensive wear that would have caused the mouthpiece to flip unevenly back and forth from the central hole to the outer ring. In addition, the pronounced collar that enclosed the central hole in the ‘four-spoked’ cheekpiece seems to have been deliberately broken off so as to enable the reassembly of the complete bit. This reassembly was achieved by reshaping one end of the bit, which resulted in an imperfect spiraled loop and producing an uneven finish with one spiral at a 90° plane relative to the other.

The defective state of the bridle bit, as well as the absence of any rings or other fittings usually associated with a headstall and reins implies that this donkey was not fully harnessed with a bridling system. This strongly suggests that, in the context of the ritual donkey burial of Tel Haror, the bridle bit had symbolic significance and its quality and functional state were of little concern.

Our conclusion that the use of the bit was symbolic is supported by examination of the teeth of the sacrificed donkey. The absence of any sign of bit wear on the lower premolars indicates that the animal was not ridden or driven with a bit for prolonged periods of time. Moreover, the young donkey was still in the process of shedding its teeth and permanent teeth were just erupting. Based on its age, the Haror donkey would probably have been too young to be a trained draught animal.

In contrast to the Haror example, most known finds of typologically similar metal bits are of uncertain date or of unknown provenance ([20]:333 [22], [25]). At the site of Tell el-Ajjul in nearby Gaza, and not far from Haror, Petrie [26] discovered a typologically similar bridle bit but unfortunately its context is not clearly defined [7]. Bits from securely dateable contexts in the Levant as well as in adjacent regions of Egypt, Anatolia, Iran and Greece are assigned to later periods and not prior to the mid-second millennium BCE [20]. Other lines of evidence suggest a use of metal bits in the third millennium BCE. For example, a ritual burial of donkeys from the Akkadian occupation at Tell Brak in northern Syria, ca. 2200 BCE, included teeth with distinctive damage as well as unusual green staining, both of which were interpreted as the result of bit wear [27]–[29]. Still, the hypothesis that these other equids had been ridden or driven with a metal bit remains conjectural ([7]:24–28). The bridle bit from Haror, though symbolic in function, remains the earliest example and the only one in the ancient Near East recorded in or near the mouth of the equid.

### Saddlebags on the Donkey's Back

**Figure 5:**
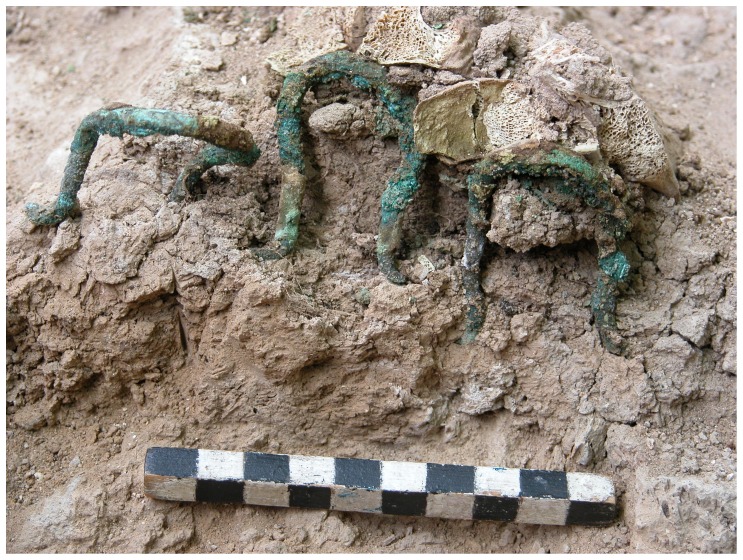
Metal fasteners of the saddlebags *in situ* (Group D: cf. **Figure 2** close –up).

Excavations of the donkey skeleton exposed a most unusual grouping of 12 corroded copper fittings placed on the upper part of the animal's rib cage (Figure 5, Figures S6 and S7; inventory numbers of Tel Haror expedition, Ben-Gurion University, Beer-Sheva 45151-45162). All of the fittings are shaped like a wide open omega sign (0.4 cm thick) with bent and slightly flattened ends. The fittings are each approximately 4.0 cm long and 4.0 cm wide and the circular section is 3.0 cm in diameter. The fittings are arranged symmetrically in four rows, 3 fittings each, ca. 2.0 cm apart and row length is 14.0-16.0 cm. Two rows designated A–B in Figure 4 were found on the upper, right side of the animal, directly on the ribcage and touching the vertebrae, whereas two additional rows (C–D) were recorded under the left pelvis (C) and behind the left femur (D), respectively. The open ends of the omega-shaped fittings of rows A–B face outwards whereas those of rows C–D are positioned the opposite way, with the curved side visible on top. This array creates a mirror image on either side of the donkey's back.

**Figure 6:**
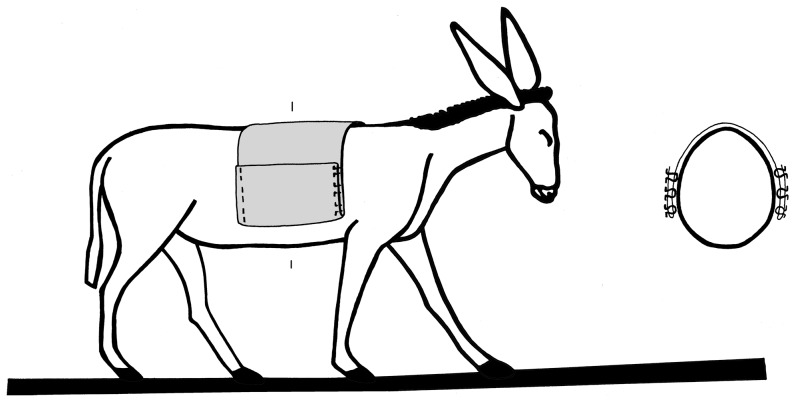
Reconstruction of the saddlebags on a donkey (from a depiction in a tomb painting at Beni Hasan, Egypt).

The location, grouping, dimensions and symmetrical placement of the fittings, leads us to suggest that they represent the remains of fasteners for saddlebags that was composed of decayed organic material, such as leather. The distances between the rows of fasteners (A–B and C–D in Figure 2) allow us to reconstruct the width of the saddle at ca. 30.0 cm. The distance between rows A and D is ca. 90.0 cm. The fact that the fasteners are distinctively positioned perpendicularly to the spine as well as on their end sections implies that the saddle sheet was folded over lengthwise. The thick margins of the double sheet seem to have been fixed together with the aid of the fasteners. The length of each row indicates that the overall size of the bag was at least 30.0×16.0 cm. However, we may presume that the metal fasteners and their respective holes were not fixed on the very edge of the bags and, hence, an extra ca. 5.0 cm must be allowed for the folded margins of the bag edge, resulting in a bag size of ca. 35.0×20.0 cm (Figure 6). The asymmetrical position of the fasteners, i.e. with open ends facing outwards on the right bag and inwards on the left bag, and their odd placement on the skeleton may be explained by the way the animal was laid down in the burial pit. Evidently, the bags were not securely strapped with a girth, but hung loosely way down the saddle. Once the donkey was lowered into the pit and laid on its left side, the bag on that side slipped to the hind part of the donkey and over the hip, while its right side bag was consequently dragged further up towards the spine of the animal.

Our hypothesis that the saddlebags were made of leather is based on the assumption that metal fasteners would not have been needed for stitching together sheets of less sturdy materials such as linen, wool or vegetal fiber, which can be sewn together with fiber cords. Metal fasteners would have been eminently suitable for stitching together thickly folded margins of leather bags.

## Discussion and Conclusions

The Tel Haror bit is the earliest known metal bit from a secure MBA context and is the only metal bit of the Bronze Age to have been recorded in context in the mouth of an equid. The bridle bit is the primary implement for directing animals by exerting pressure on sensitive parts of their head [7], [20]. Thus, the development of the metal bridle bit and its use during the MBA in the Levant provided an effective device for directional control and swift maneuverability and marks a significant technological leap [23]–[24], [30]–[31].

The discovery at Tel Haror of a metal bridle bit, which was essential for controlling and steering effectively a horse team traveling at speed [31], also bears on the early history of the light chariot in the Levant and Egypt and its eventual incorporation into the military organization [7], [25]. Evidence from historical and iconographic sources indicates that light, horse-drawn chariots (primarily for military use) were already known in the ancient Near East by the 18^th^ century BCE [30]. The Tel Haror bridle bit provides the first archaeological evidence found *in situ* in a clear MBA context dated to the periods between the 17^th^ or mid-16^th^ centuries BCE.

Evidence from texts and iconographic depictions from the Near East shows that donkeys were ridden or driven with a neck rope, nose ring or nose band [32]. Harnessing of a donkey with a bridling system was so far unknown. The observation that the Haror bit was defective and hardly serviceable is a strong indication that the horse bit was inserted *ad hoc* either preceding or following its ritual butchering. Texts and iconographic representations from Syria and Anatolia indicate that equids participated in ritual ceremonies, e.g., carrying the images or statues of deities or pulling the carts or wagons of these deities in processions as well as reared and stabled in temple domains [27]–[28], [32]–[34].

The Tel Haror saddlebags are the earliest archaeological evidence for this equid-borne pannier in the Levant and Egypt prior to the Iron Age. The presence of bags on either side of the saddle as well as their position on the donkey rules out the possibility that the saddlebags were used for riding. So far, in the Levant, riding saddles are not documented before the Neo-Assyrian period, i.e., in the 8^th^ century BCE. Numerous representations of donkey riders in Bronze Age Levantine and Egyptian art show that they do not use a proper riding saddle but a pack saddle ([35]:50).

The Tel Haror saddlebags may have been crafted specifically for the occasion of the donkey burial in the temple precinct. This suggestion is supported by the distinctively small sized bags, 35.0×20.0 cm, and the ritual context of the specimen. The saddlebags may have been used to carry certain valuable items.

References to saddlebags occur often in ancient texts of economic matters that deal with essential equipment for the use of donkeys as pack animals [36]. To date, our knowledge of Bronze Age saddlery is largely based on Egyptian tomb paintings of the late First Intermediate and Middle Kingdom periods (21^st^–19^th^ centuries BCE), as well as various MBA glyptics [37]. In the Egyptian paintings, donkeys are depicted with sacks lashed onto sizable pack saddles or mats, as well as bags or double-bags mounted on mats or directly over the animal's back. These were made of dyed woolen fabrics or woven vegetal fibers of local plants. Their size, ca. 70.0/80.0×50.0/60.0 cm, is more than twice as large as the Tel Haror bags ([38]:82–83; [39]: Plate 31; [40]: 64–67).

Thus far, the donkey burial in the temple courtyard, in the heart of the sacred precinct at Tel Haror is the only known example of a sacrificed donkey harnessed symbolically with a horse bit in a ritual context. Whereas the sacrificed donkey signifies an accepted ritual mode in contemporary ‘Amorite’ tradition, the metal bit is not a usual component of its ritual paraphernalia. Rather, the symbolic significance of the horse bit may be related to the technological innovation related to chariotry [30]. Moreover, bridle bits, like elite weapon types, were symbols of status, much like the horses and chariots.

The association of the Tel Haror donkey sacrifice with a bridle bit and saddlebags may suggests that, prior to the killing, a ceremony was performed in the temple courtyard. The deposition of a fully articulated donkey testifies that the animal was not consumed and that the ceremony may have been concluded with a ritual meal or a feast. This conclusion is supported by the numerous bones of butchered sheep and goat discovered above the donkey's carcass. Further evidence for the practice of the Amorite rituals at Tel Haror, is the discovery of articulated and ritually killed juvenile dogs in sacred repositories (*favissae*). The ritual slaughter of young dogs is likewise recorded in treaty documents in the Mari archive.

The donkey of Tel Haror with its bridle bit and saddlebags reflects significant social, economic and technological developments related to the use of equids in the Near East. The combined textual, iconographic and archaeological record from the Levant and Egypt testifies categorically to the symbolic prominence of equids during the Bronze Age and particularly in the course of the MBA. During this period of intensified economic activities, involving merchant donkey caravans in the 19^th^–17^th^ century BCE donkeys became a major economic asset in this region and a source of its prosperity. The rich archival record from Mari and other ‘Amorite’ centers in Syria (ca. 18^th^–17^th^ century BCE) provides ample data on extensive trading networks and donkey caravans that operated across the Levant and Anatolia, respectively [41]–[42]. Similarly, contemporary Egyptian inscriptions document large-scale expeditions, including hundreds of pack donkeys destined for the mining sites in the eastern desert and southern Sinai [43]–[46]. In addition, the pictorial and figurative record depicts equids (donkeys, horses, onagers and hybrids) in various transport tasks of both a mundane and elite character [40], the latter evincing the high social status of donkey riding.

Judging from these rich textual and iconographic records the donkey was clearly a cultural-ideological marker to which high symbolic value was attached. This reflects the centrality of the donkey as a pack animal in societies for which caravans played a pivotal economic role. Indeed, many textual references and iconographic representations reflect the high social status of donkey riders. Donkey riding was thus a privilege enjoyed by royalty and other high-ranking personalities [42], [47].

## Methods

All necessary permits were obtained for the described field study. Excavation was directed by E.D. Oren, P. Nahshoni and G. Bar-Oz on behalf of Ben-Gurion University of the Negev (Israel Antiquities Authority license no. G-61/2010).

The bones of the excavated skeletons were fragile and badly preserved and most long bones, the pelvis and the skull were broken *in situ*. Therefore during excavation we removed the sediment adjacent to and surrounding the bones to achieve full exposure of bones. This allowed also measuring them while still *in situ*.

Identification of the excavated skeleton as a donkey (*Equus asinus*) was first carried out by J. Klenck and R.H. Meadow and J. Clutton-Brock and was based on the enamel patterns of the cheek teeth (following [3], [48]). Clutton-Brock also examined the teeth and determined its age (Clutton-Brock, personal communication 2010). This identification was further approved during the 2010 season by the size and proportions of limb bones [49], [50]. Each of the bones was examined under a 10× Olympus stereomicroscope with an oblique light source for bone surface modifications (butchery, burning, carnivore puncture, scoring, and digestion) and pathological bone alteration [51], [52]. The age of the excavated specimens was analyzed during the 2010 season using tooth wear [53], [54] and epiphyseal closure data [53].

## Supporting Information

Figure S1 **Tell Haror excavation areas.**
(TIF)

Figure S2 **The Sacred Precinct in Area K; (1) the Syrian-type temple (L8630); (2,3) courtyards (L8530, 8361); (4,5) altars (L8269, 8705); (6) the favissa area (L8430); (7) the storehouse (L8888); (8) an offering room with benches (L8094); (9) the circular offering installation (L8740) and (10) the donkey burial pit (L8877).**
(TIF)

Figure S3 **Section the circular offering installation.**
(TIF)

Figure S4 **The donkey mandible and ceramic vessels found above the main donkey burial (L8746).**
(TIF)

Figure S5 **Drawing of bronze bit.**
(TIF)

Figure S6 **Fastener A2 of the saddlebag.**
(TIF)

Figure S7 **Drawing of fastener A2.**
(TIF)

Table S1 **Measurements of Haror donkeys.**
(DOCX)
