# Supplementary figures and images for: Symbolic Metal Bit and Saddlebag Fastenings in a Middle Bronze Age Donkey Burial
Source: PLoS One. 2013 Mar 6;8(3):e58648. doi: 10.1371/journal.pone.0058648 (PMC3590166; doi:10.1371/journal.pone.0058648)

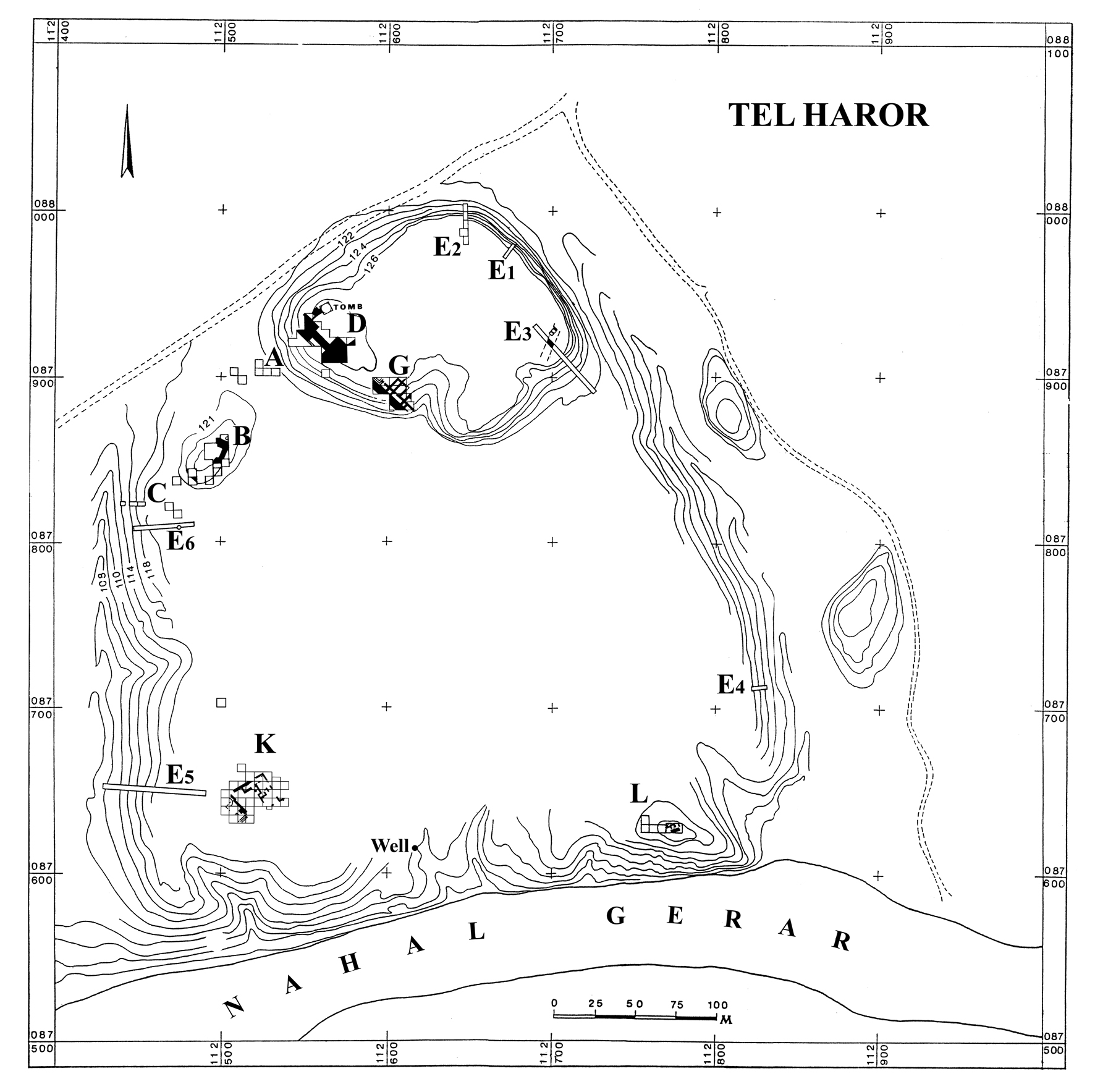

Supplement: Figure S1 — Tell Haror excavation areas. (TIF) [file pone.0058648.s001.tif]

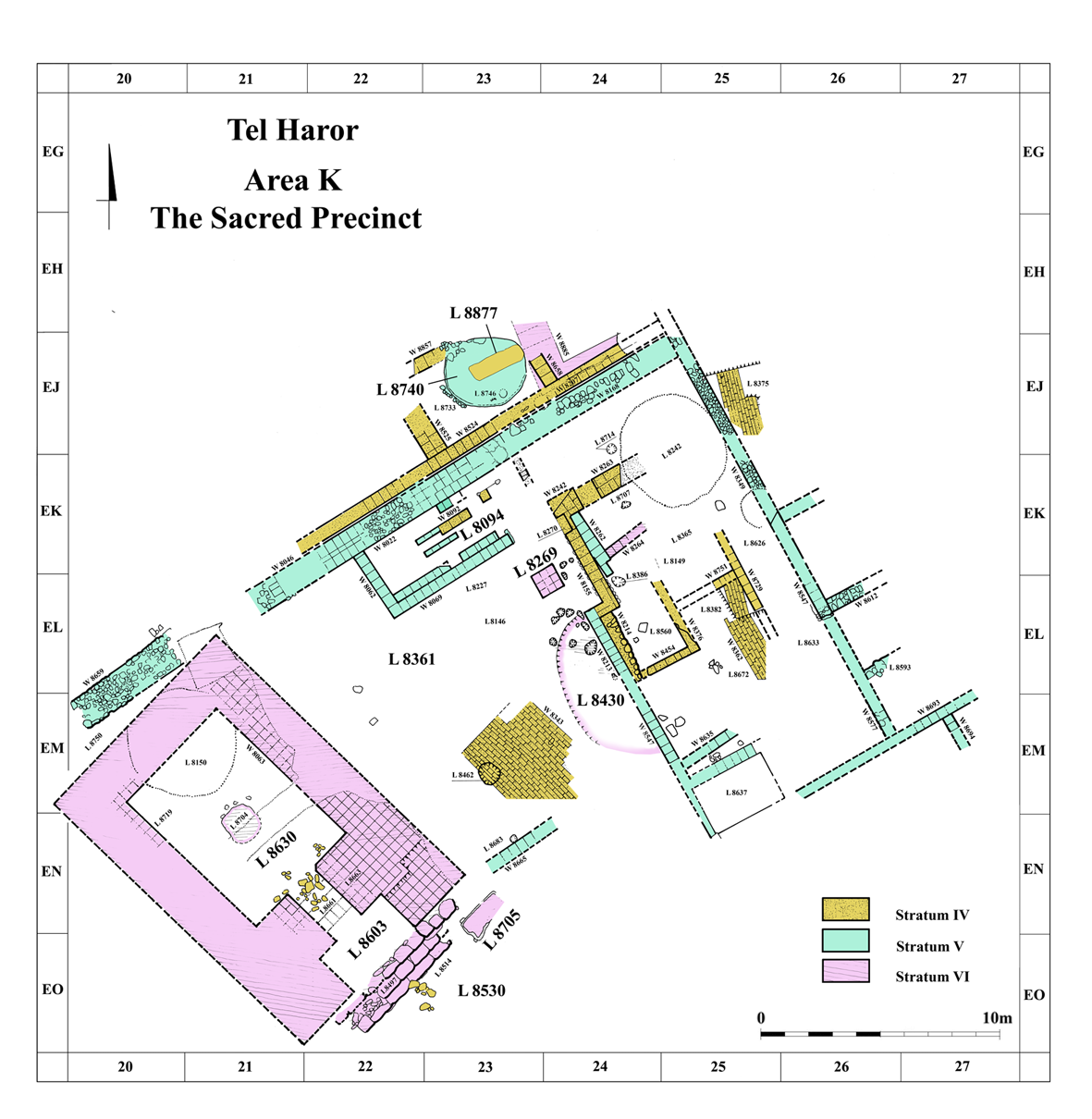

Supplement: Figure S2 — The Sacred Precinct in Area K; (1) the Syrian-type temple (L8630); (2,3) courtyards (L8530, 8361); (4,5) altars (L8269, 8705); (6) the favissa area (L8430); (7) the storehouse (L8888); (8) an offering room with benches (L8094); (9) the circular offering installation (L8740) and (10) the donkey burial pit (L8877). (TIF) [file pone.0058648.s002.tif]

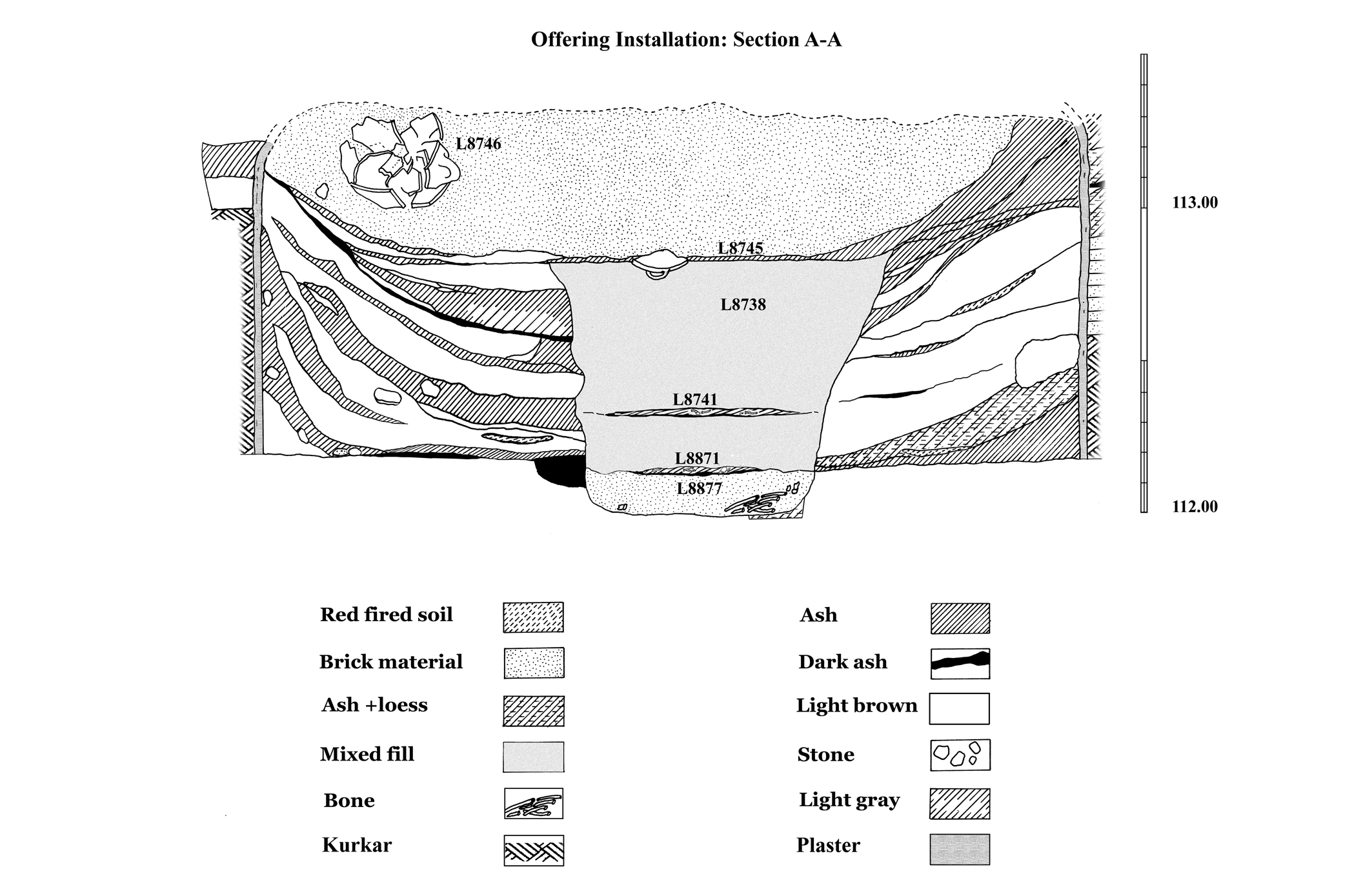

Supplement: Figure S3 — Section the circular offering installation. (TIF) [file pone.0058648.s003.tif]

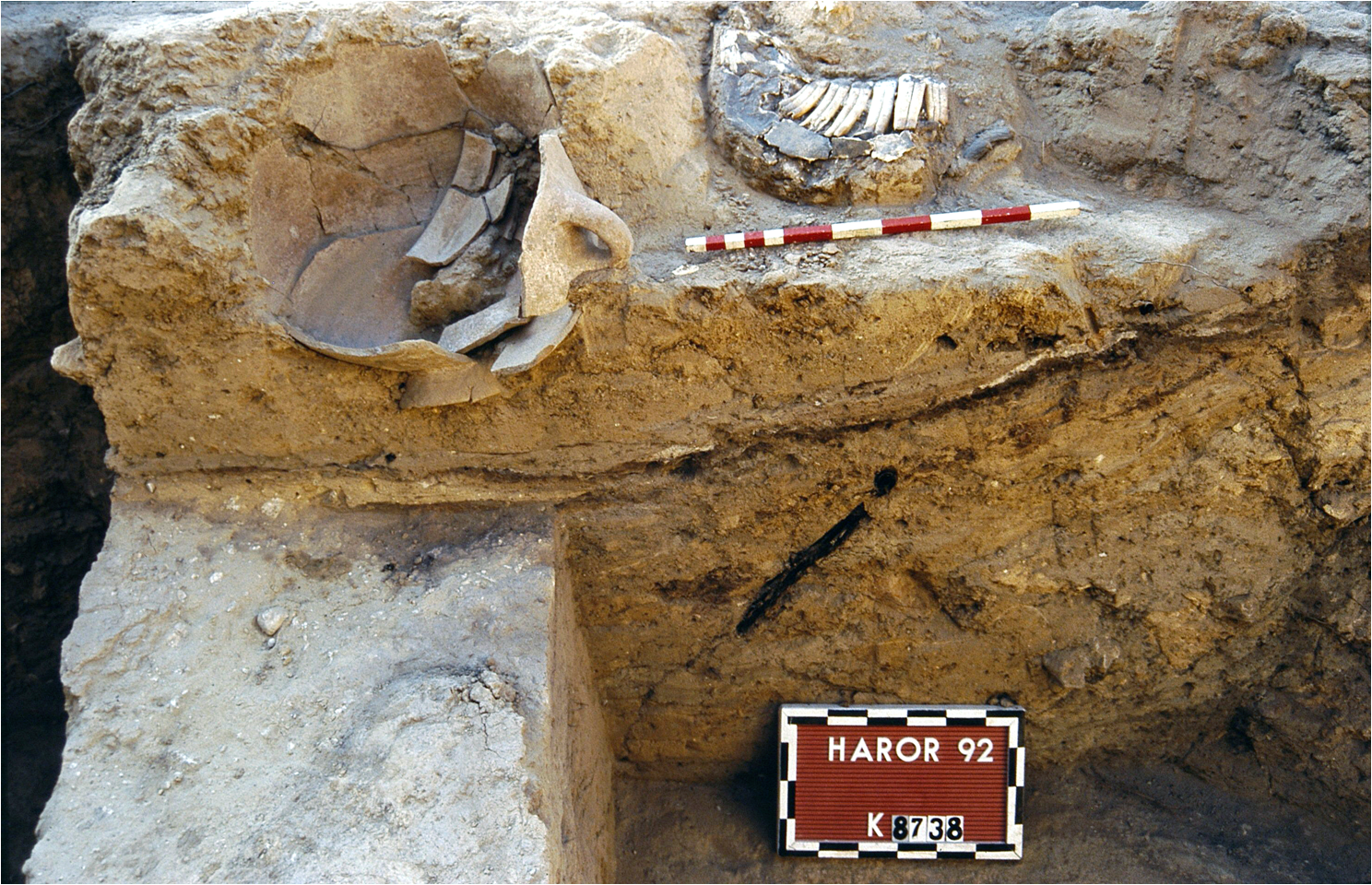

Supplement: Figure S4 — The donkey mandible and ceramic vessels found above the main donkey burial (L8746). (TIF) [file pone.0058648.s004.tif]

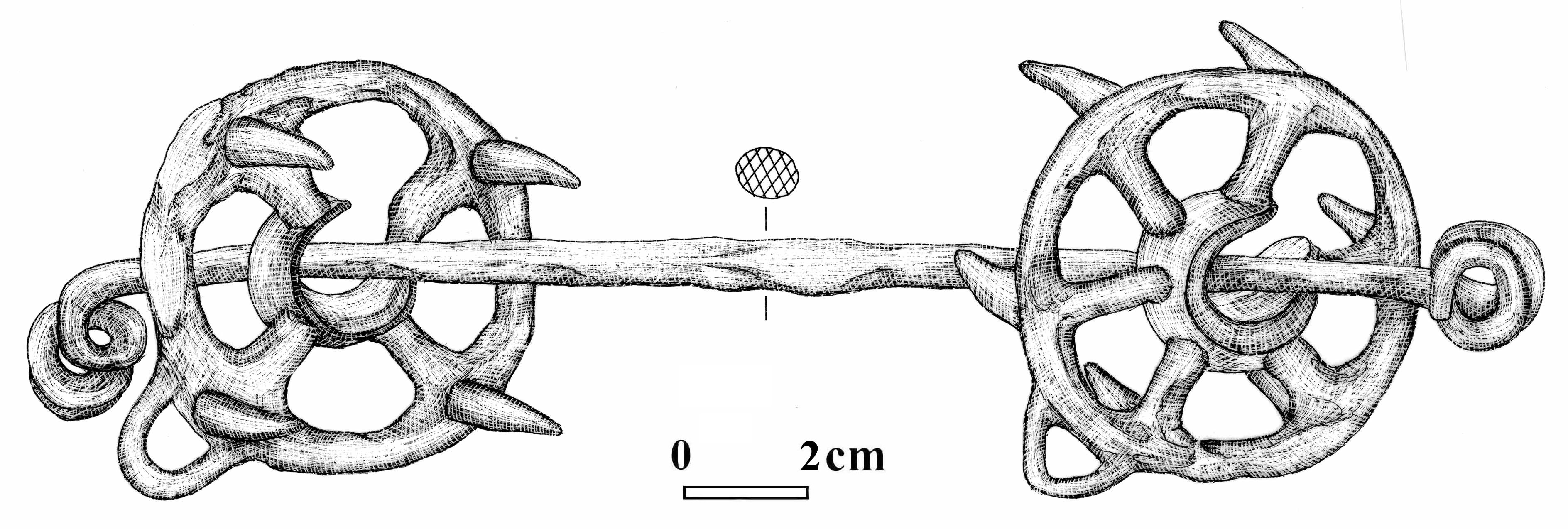

Supplement: Figure S5 — Drawing of bronze bit. (TIF) [file pone.0058648.s005.tif]

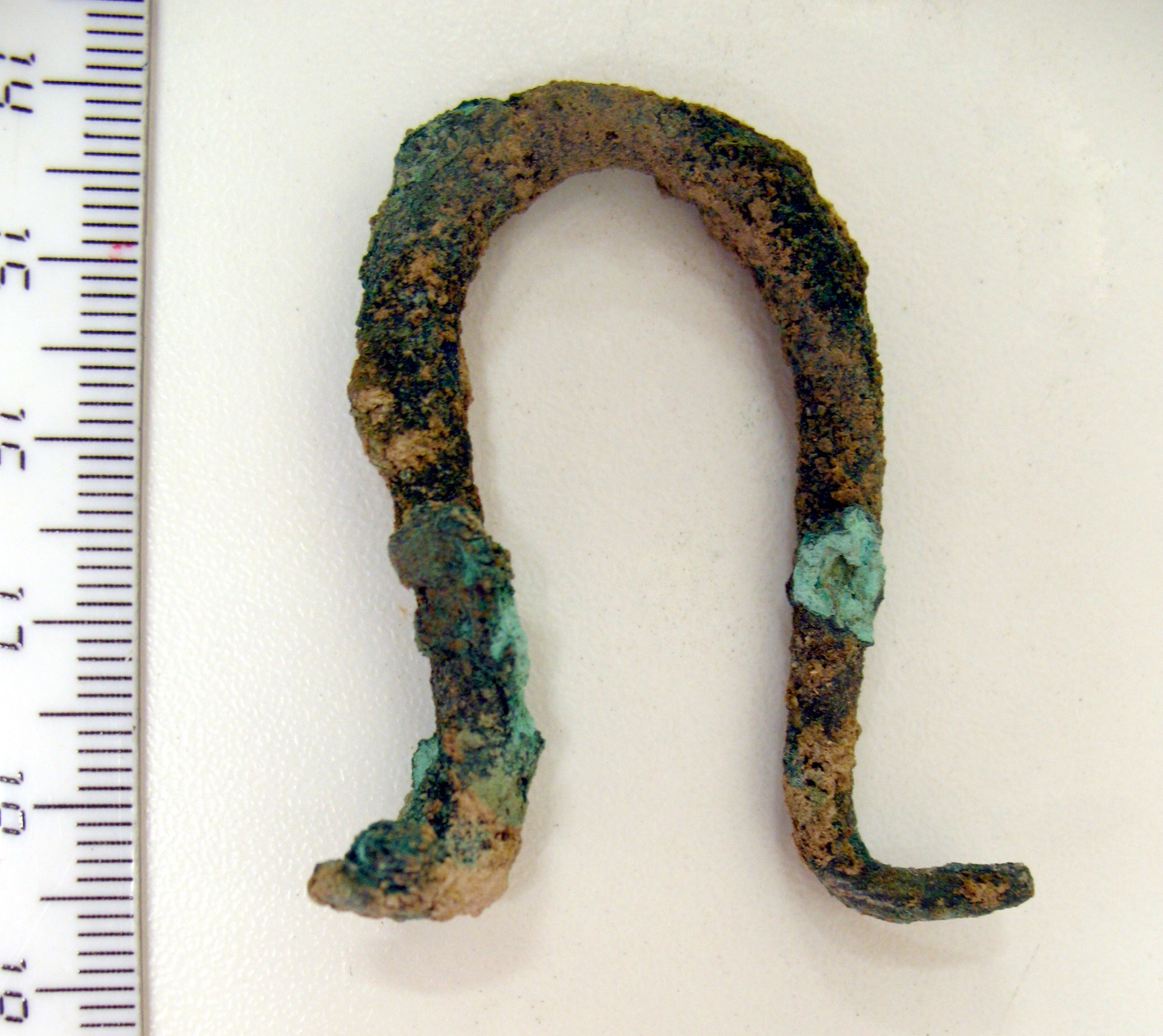

Supplement: Figure S6 — Fastener A2 of the saddlebag. (TIF) [file pone.0058648.s006.tif]

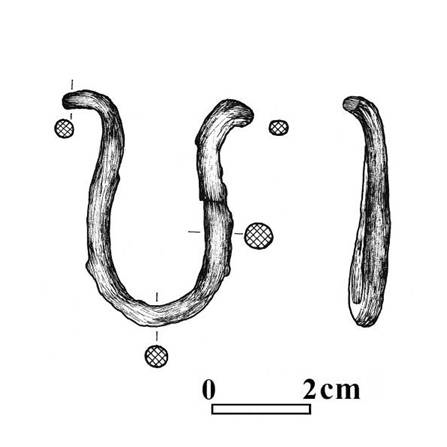

Supplement: Figure S7 — Drawing of fastener A2. (TIF) [file pone.0058648.s007.tif]
