# Supplementary material for: Symbolic Metal Bit and Saddlebag Fastenings in a Middle Bronze Age Donkey Burial
Source: PLoS One. 2013 Mar 6;8(3):e58648. doi: 10.1371/journal.pone.0058648 (PMC3590166; doi:10.1371/journal.pone.0058648)
Supplement: Table S1 — Measurements of Haror donkeys. (DOCX) [file pone.0058648.s008.docx]

**Table S1.**


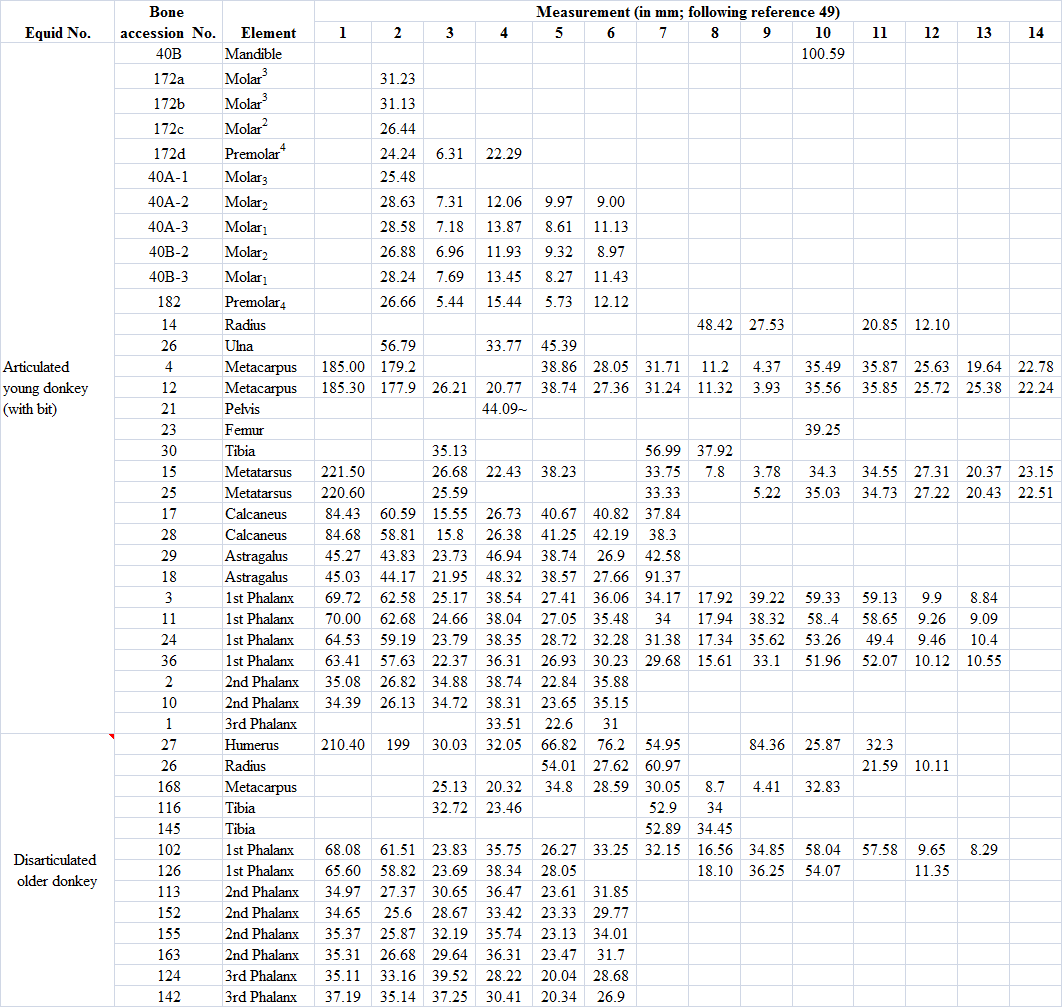


**Measurements Index:**

*Mandible:* 10 - height of the jaw posterior to M3

*Upper Tooth:* 2 - occlusal length; 3 - occlusal length of the protocone; 4 - occlusal breadth

*Lower Tooth:* 2 - tooth length; 3 - length of the preflexid; 4 - length of the double knot; 5 - length of the postflexid; 6 - maximal breadth

*Humerus:* 1 - maximal length; 2 - maximal length from caput; 3 - minimal breadth; 4 Diameter perpendicular to, and at the level of 3; 5 - proximal maximal breadth; 6 - proximal depth at the level of the median tubercule; 7 - maximal breadth of the trochlea; 9 - distal maximal depth; 10 - maximal trochlear height (medial); 11 - Minimal trochlear height (in the middle); 12 - trochlear height at the sagittal crest (near the condyle)

*Radius:* 5 - proximal articular breadth; 6 - proximal articular depth; 7 - proximal maximal breadth; 8 - distal articular breadth; 9 - distal articular depth; 10 - distal maximal breadth; 11- breadth of the radial condyle; 12 - breadth of the ulnar condyle

*Ulna:* 2 - length of the olecranon; 4 - minimal depth of the olecranon; 5 - depth across the processus anconaeus

*Femur:* 10 - maximal depth of caput femoris

*Tibia:* 3 - minimal breadth; 4 - minimal depth of the diaphysis (more or less at the level of 3); 7 - distal maximal breadth; 8 - distal maximal depth

*Metacarpus:* 1 - maximal length; 2 - medial length; 3 - minimal breadth (near the middle of the bone); 4 - depth of the diaphysis at level of 3; 5 - proximal articular breadth; 6 - proximal articular depth; 7 - maximal diameter of the articular facet for the third carpal; 8 - diameter of the anterior facet for the fourth carpal; 9 - diameter of the articular facet for the second carpal; 10 - distal maximal supra-articular breadth; 11 - distal maximal articular breadth; 12 - distal maximal depth of the keel; 13 - distal minimal depth of the lateral condyle; 14 - distal maximal depth of the medial condyle

*Metatarsus:* 1 - maximal length; 2 - medial length; 3 - minimal breadth (near the middle of the bone); 4 - depth of the diaphysis at level of 3; 5 - proximal articular breadth; 6 - proximal articular depth; 7 - maximal diameter of the articular facet for the third tarsal; 8 - diameter of the anterior facet for the fourth tarsal; 9 - diameter of the articular facet for the second tarsal; 10 - distal maximal supra-articular breadth; 11- distal maximal articular breadth; 12- distal maximal depth of the keel; 13 - distal minimal depth of the lateral condyle; 14 - distal maximal depth of the medial condyle

*Calcaneus:* 1 - maximal length; 2 - length of the proximal part; 3 - minimal breadth; 4 - proximal maximal breadth; 5 - proximal maximal depth; 6 - distal maximal breadth; 7 - distal maximal depth

*Astragalus:* 1 - maximal length; 2 - maximal diameter of the medial condyle; 3 - breadth of the trochlea (at the apex of each condyle); 4 - maximal breadth; 5 - distal articular breadth; 6 - distal articular depth; 7 - maximal medial depth

*1st Phalanx:* 1 - maximal length; 2 - anterior length; 3 - minimal breadth; 4 - proximal breadth; 5 - proximal depth; 6 - distal breadth at the tuberosities; 7 - distal articular breadth; 8 - distal articular depth; 9 - minimal length of the trigonum phalanges; 10 - medial supratuberosital length; 11 - lateral supratuberosital length; 12 - medial infratuberosital length; 13 - lateral infratuberosital length

*2nd Phalanx:* 1 - maximal length; 2 - anterior length; 3 - minimal breadth; 4 - proximal maximal breadth; 5 - proximal maximal depth; 6 - distal articular maximal breadth;

*3rd Phalanx:* 1 - length from the posterior edge of the articular surface to the tip of the phalanx; 2 - anterior length; 3 - maximal breadth; 4 - articular breadth; 5 - articular depth; 6 - maximal height
